# The Role of Metformin in Controlling Oxidative Stress in Muscle of Diabetic Rats

**DOI:** 10.1155/2016/6978625

**Published:** 2016-08-04

**Authors:** Danielle Diniz Vilela, Leonardo Gomes Peixoto, Renata Roland Teixeira, Nathalia Belele Baptista, Douglas Carvalho Caixeta, Adriele Vieira de Souza, Hélen Lara Machado, Mariana Nunes Pereira, Robinson Sabino-Silva, Foued Salmen Espindola

**Affiliations:** ^1^Institute of Genetics and Biochemistry, Federal University of Uberlandia, 38400-902 Uberlandia, MG, Brazil; ^2^Department of Physiology, Institute of Biomedical Sciences, Federal University of Uberlandia, 38400-902 Uberlandia, MG, Brazil

## Abstract

Metformin can act in muscle, inhibiting the complex I of the electron transport chain and decreasing mitochondrial reactive oxygen species. Our hypothesis is that the inhibition of complex I can minimize damage oxidative in muscles of hypoinsulinemic rats. The present study investigated the effects of insulin and/or metformin treatment on oxidative stress levels in the gastrocnemius muscle of diabetic rats. Rats were rendered diabetic (D) with an injection of streptozotocin and were submitted to treatment with insulin (D+I), metformin (D+M), or insulin plus metformin (D+I+M) for 7 days. The body weight, glycemic control, and insulin resistance were evaluated. Then, oxidative stress levels, glutathione antioxidant defense system, and antioxidant status were analyzed in the gastrocnemius muscle of hypoinsulinemic rats. The body weight decreased in D+M compared to ND rats. D+I and D+I+M rats decreased the glycemia and D+I+M rats increased the insulin sensitivity compared to D rats. D+I+M reduced the oxidative stress levels and the activity of catalase and superoxide dismutase in skeletal muscle when compared to D+I rats. In conclusion, our results reveal that dual therapy with metformin and insulin promotes more benefits to oxidative stress control in muscle of hypoinsulinemic rats than insulinotherapy alone.

## 1. Introduction

Oxidative stress reflects an imbalance between reactive oxygen species (ROS) production and the biological systems ability to detoxify the reactive intermediates. The antioxidant defense includes both enzymatic and nonenzymatic mechanisms, which protect the cell against ROS [1, 2]. An increase in oxidative stress is associated with hyperglycemia, development, and progression of diabetes complications [3, 4]. This condition can lead to lipid peroxidation in muscle cell membranes, which contributes to the development of insulin resistance [5, 6].

Metformin, a biguanide derivate, is mainly used to treat patients with type 2 diabetes mellitus (T2D) [7]. Metformin activates intracellular signaling pathways in response to cellular energy changes in skeletal muscle [8, 9], which improves glucose uptake [10]. Several studies have shown that the primary effect of metformin is the inhibition of mitochondrial complex I (NADH: ubiquinone oxidoreductase) [11–13]. Mitochondrial complex I may contribute substantially to the cellular ROS production [14]. It is well documented that a blockage this complex leads to decreased production of reactive species, due to reduced transport of electrons from NADH plus H^+^ [15, 16]. Therefore, evidence suggests that metformin reduces endogenous ROS mitochondrial levels [17, 18].

Despite metformin being widely used in T2D treatment [7, 19], recent clinical studies evaluated dual therapy with insulin and metformin for type 1 diabetic (T1D) patients [20, 21]. This dual therapy showed that glycemic control was better or similar to insulin therapy alone. We do not known studies that verified the role of metformin associated with insulin compared to insulin therapy alone in oxidative stress control in gastrocnemius muscle of streptozotocin-diabetic rats.

Consider that altered oxidative stress/antioxidant status is related to T1D complications [22, 23] and metformin action in the decrease of mitochondrial ROS. The purpose of the present study was to verify the action of metformin combined with insulin in oxidative stress control of streptozotocin-diabetic rats. To address this questions, we investigated the antioxidant defense system enzymatic (glucose-6-phosphate dehydrogenase (G6PDH), glutathione reductase (GR), glutathione peroxidase (Gpx), catalase (CAT), and superoxide dismutase (SOD)) and nonenzymatic (reduced glutathione (GSH)) in gastrocnemius muscle of diabetic rats. We also evaluated total antioxidant capacity and lipid peroxidation in gastrocnemius muscle of diabetic rats treated with metformin and/or insulin. Furthermore, the glycemic control and insulin resistance were analyzed under metformin and/or insulin therapy.

## 2. Materials and Methods

### 2.1. Animals

Male Wistar rats (approximately 7 weeks old and weighing 200–270 g) were kept in controlled conditions (22 ± 1°C, humidity 60%  ± 5, and 12 hour light-dark cycles) with standard diet and water* ad libitum*. All experimental procedures were approved and conducted by the Brazilian Society of Laboratory Animal Science and the Ethics Committee for Animal Research of the Federal University of Uberlandia, Brazil (CEUA/UFU: Approval number 107/14).

### 2.2. Diabetes Induction

The rats were subjected to 24-hour starvation and given an intraperitoneal injection of streptozotocin (STZ, 45 mg/kg body weight, dissolved in 0.01 M sodium citrate buffer, pH 4.5) to induce diabetes. Nondiabetic (ND) animals received the same volume of citrate buffer. Blood glucose was determined with tail blood, using test strips (Accu-Chek Performa) 10 days after the STZ injection. Rats with a fasting glucose level > 250 mg/dL were considered diabetic.

### 2.3. Groups and Treatment

Diabetic animals were randomly divided into 4 groups (*n* = 6-7 animals per group): untreated diabetics (D), diabetic treated with insulin (D+I), diabetic treated with metformin (D+M), and diabetic treated with insulin and metformin (D+I+M). After diabetes characterization, the D+I and D+I+M rats were submitted to a seven-day treatment with insulin (Novolin N Human Insulin -NPH) at a dose of 3 units per day (1 U at 8:00 a.m. and 2 U at 5:00 p.m. subcutaneously). The metformin (metformin hydrochloride, Merck) was diluted with filtered water and D+M and D+I+M animals received 500 mg/kg body weight at 6:00 p.m., by oral gavage during seven days. The ND rats received filtered water by oral gavage.

The last dose of insulin was administered at 5:00 p.m. and metformin at 6:00 p.m. the day before samples collection. On the next day, the* ad libitum* fed rats were anesthetized at 8:00 a.m. with sodium thiopental (80 mg/kg body weight, i.p.; 13-14 hours after the last dose of metformin and insulin treatment). Then, glycemia and body weight were evaluated. Thus, the observed effects on glycemia and oxidative stress markers are associated with the prolonged treatment effect.

### 2.4. Sample Collection and Tissue Preparation

The gastrocnemius muscle was chosen because it contains more variety of type II fibers, which are glycolytic in nature [24]. The muscle was quickly removed, washed using normal saline (NaCl 0.9%), and frozen by immersing it in liquid nitrogen. For oxidative stress markers and western blotting analyses, muscle tissue was homogenized in a phosphate buffer (1 : 10 w/v, pH 7.4). The homogenates were centrifuged at 15,000 ×g for 10 min at 4°C and total protein concentration in the supernatant samples was measured, following the Bradford assay [25].

### 2.5. Intravenous Insulin Tolerance Test (kITT)

Tail blood samples were collected in* ad libitum* fed animals before (0 min) and 4, 8, 12, and 16 min after an i.v. injection of regular insulin (0.75 U/kg BW; Humulin R). The constant rate for blood glucose disappearance during insulin tolerance test (kITT) was calculated based on the linear regression of the Naperian logarithm of blood glucose concentrations (test strips, Accu-Chek Performa). The tests were performed between 08:00 to 10:00.

### 2.6. Oxidative Stress Markers Analysis

#### 2.6.1. Lipid Peroxidation (TBARS)

Lipid peroxidation was measured in the gastrocnemius muscle by reacting malondialdehyde in sample (MDA) and thiobarbituric acid (0.67% TBA). The organic-phase was evaluated with a fluorometer at 515 nm excitation and 553 nm emission. MDA standard curve allowed for the quantification of the this compound in the samples, by linear regression [26].

#### 2.6.2. Total Antioxidant Capacity (FRAP)

Antioxidants present in the samples reduced Fe^+3^ (ferric chloride solution 20 mM) to Fe^+2^, which is chelated by 2,4,6-tri(2pyridyl)-s-triazine (TPTZ 10 mM), forming the complex Fe^+2^-TPTZ, which has an intense blue color. This complex was evaluated in the spectrophotometer at 593 nm.

#### 2.6.3. Glucose 6-Phosphate Dehydrogenase Activity (G6PDH)

The activity of glucose 6-phosphate dehydrogenase was monitored by the production of NADPH with a consequent increase in absorbance at 340 nm. The reading was performed in a microplate, where the samples were incubated with Tris-HCl buffer (100 mM, pH 7.5), magnesium chloride (MgCl_2_ 2 M), NADP + (0.5 mM), and glucose 6-phosphate (1 mM). The kinetic reading was taken for 10 minutes [27].

#### 2.6.4. Glutathione Reductase Activity (GR)

The GR assay to quantify the enzymatic activity was registered by a decrease in the concentration of NADPH in the samples, using GR buffer (200 mM sodium phosphate pH 7.5, 6.3 mm EDTA) and NADPH. The kinetic reading was performed at 340 nm for 10 minutes [28].

#### 2.6.5. Glutathione Peroxidase Activity (Gpx)

To measure the activity of glutathione peroxidase, the homogenate was incubated with Gpx buffer (100 mM potassium phosphate with 1 mM EDTA pH7.7), 40 mM sodium azide, GSH (diluted in 5% metaphosphoric acid), GR (Gpx diluted in buffer), NADPH (diluted with sodium bicarbonate 5%), and 0.5 mM tert-butyl. The decay of NADPH concentration was evaluated for 10 minutes in a spectrophotometer, at 340 nm [29].

#### 2.6.6. Reduced Glutathione (GSH)

The protein content of the samples was initially precipitated by metaphosphoric acid (MPA) in the ratio 1 : 1 (homogenate/MPA). The samples were centrifuged at 7000 ×g for 10 minutes and the supernatant was used for the measurements. A standard curve of GSH (0.001–0.1 mM) was made to quantify GSH in the samples using a linear regression. GSH reacts with ortho-phthalaldehyde (OPT 1 mg/mL methanol) diluted in sodium phosphate monobasic buffer (0.1 M) and EDTA (0.005 M). The reading was done in a fluorometer with excitation 350 nm and emission 420 nm [30].

### 2.7. Western Blotting

Aliquots of supernatant samples were solubilized in electrophoresis sample buffer, containing 100 mM Tris-HCl, pH 8.0, and 25% glycerol. Twenty micrograms of protein was electrophoresed and transferred to nitrocellulose membrane using Tris-glycine buffer [31]. The membranes were blocked with 5% dried milk in PBS-T (50 mM Tris-HCl, pH 8.0, 150 mM NaCl, and 0.05% Tween 20) and then incubated with catalase and SOD primary antibodies (0.1 *µ*g/mL). The samples were incubated with a peroxidase-conjugated anti-rabbit IgG (diluted 1 : 5000). Antibodies bound to the membranes were visualized by chemiluminescence. The intensity of the protein bands was analyzed by ImageQuantTL software and results were expressed by densitometry.

### 2.8. Statistical Analysis

All values are presented as mean ± SEM. Blood glucose was analyzed by a one-way analysis of variance (ANOVA), followed by Dunnett's Multiple Comparison as a posttest. The analysis of oxidative stress biomarkers was performed by ANOVA, followed by Tukey Multiple Comparison as a posttest. All analyses were performed in the program GraphPad Prism (GraphPad Prism version 4.00 for Windows; GraphPad Software, San Diego, CA, USA). *p* < 0.05 was considered as significant.

## 3. Results

**Figure 1:**
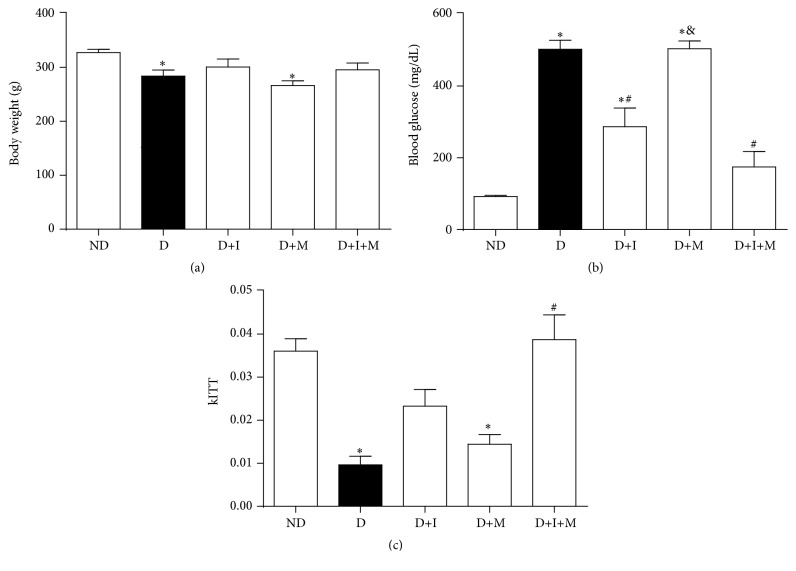
Body weight, glycemic control, and insulin tolerance test of nondiabetic and diabetic rats treated with insulin and/or metformin. Body weight (a), blood glucose level (b), and intravenous insulin tolerance test (kITT) (c). Nondiabetic (ND), diabetic (D), diabetic treated with insulin (D+I), diabetic treated with metformin (D+M), and diabetic treated with insulin and metformin (D+I+M). ^*∗*^ *p* < 0.05 versus ND, ^#^ *p* < 0.05 versus D, and ^&^ *p* < 0.05 versus D+I.

Figure 1 shows the body weight, glycemic control, and insulin tolerance test of diabetic rats treated with insulin and/or metformin. The body weight did not change among the diabetic groups, whereas D and D+M showed a decrease in body weight compared to ND rats (Figure 1(a)). As expected, plasma glucose levels increased (*p* < 0.05) in D compared to ND rats. D+I and D+I+M rats showed similar blood glucose levels and a decrease in this parameter compared to D rats (*p* < 0.05). Blood glucose levels were similar in D+M compared to D rats (Figure 1(b)). The insulin tolerance test showed a decrease in insulin sensitivity in D compared to ND rats (*p* < 0.05). In addition, D+I+M rats showed an increase in insulin sensitivity compared to D rats (*p* < 0.05) and presented similar results compared to D+I rats (*p* > 0.05) (Figure 1(c)).

**Figure 2:**
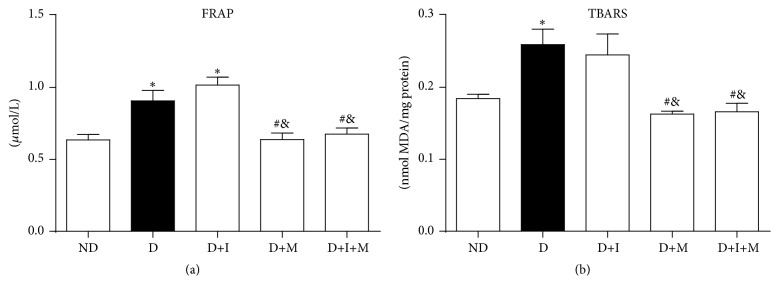
Analysis of biomarkers of oxidative stress in gastrocnemius muscle of nondiabetic and diabetic rats treated with insulin and/or metformin. Total antioxidant capacity (FRAP) (a) and lipid peroxidation (TBARS) (b). Nondiabetic (ND), diabetic (D), diabetic treated with insulin (D+I), diabetic treated with metformin (D+M), and diabetic treated with insulin and metformin (D+I+M). ^*∗*^ *p* < 0.05 versus ND, ^#^ *p* < 0.05 versus D, and ^&^ *p* < 0.05 versus D+I.

Oxidative stress analyses in gastrocnemius muscle of diabetic rats are shown in Figure 2. Total antioxidant capacity (FRAP) and lipid peroxidation (TBARS) increased (*p* < 0.05) in D compared to ND rats. In addition, D+I rats did not show any difference in the total antioxidant capacity and lipid peroxidation compared to D rats. On the other hand, D+I+M and D+M rats showed a decrease in these parameters compared to D and D+I rats (*p* < 0.05) (Figure 2).

**Figure 3:**
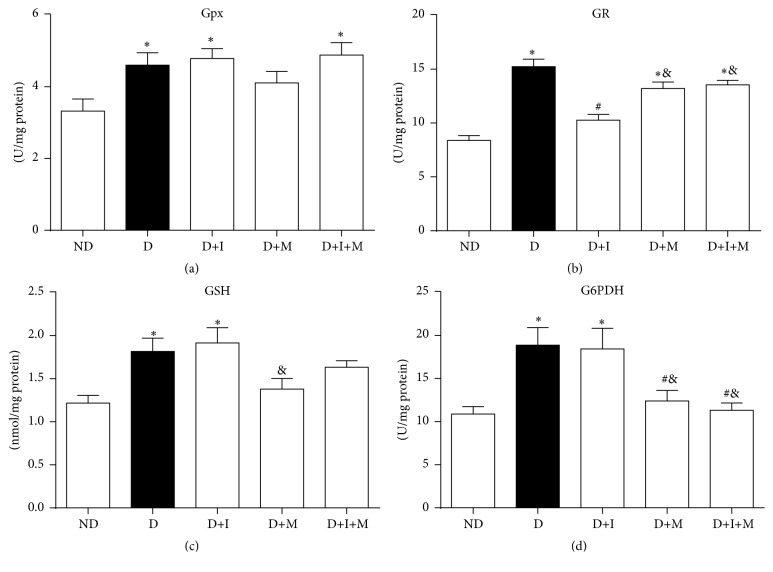
Glutathione antioxidant defense system in gastrocnemius muscle of nondiabetic and diabetic rats treated with insulin and/or metformin. Glutathione peroxidase activity (Gpx) (a), glutathione reductase activity (GR) (b), reduced glutathione (GSH) (c), and glucose-6-phosphate dehydrogenase activity (G6PDH) (d). Nondiabetic (ND), diabetic (D), diabetic treated with insulin (D+I), diabetic treated with metformin (D+M), and diabetic treated with insulin and metformin (D+I+M). ^*∗*^ *p* < 0.05 versus ND, ^#^ *p* < 0.05 versus D, and ^&^ *p* < 0.05 versus D+I.

Figure 3 shows the glutathione defense system in the gastrocnemius muscle of diabetic rats treated with insulin and/or metformin. The activities of Gpx, GR, and G6PDH enzymes and GSH concentration increased in D compared to ND rats (Figures 3(a)–3(d)). Gpx and G6PDH activities and GSH concentration did not change in D+I compared to D rats (*p* < 0.05) (Figures 3(a), 3(c), and 3(d)). However, GR activity in gastrocnemius muscle decreased in D+I compared to D rats (*p* < 0.05) (Figure 3(b)). In addition, G6PDH activity decreased (*p* < 0.05) in D+M and D+I+M compared to D and D+I rats (Figure 3(d)).

**Figure 4:**
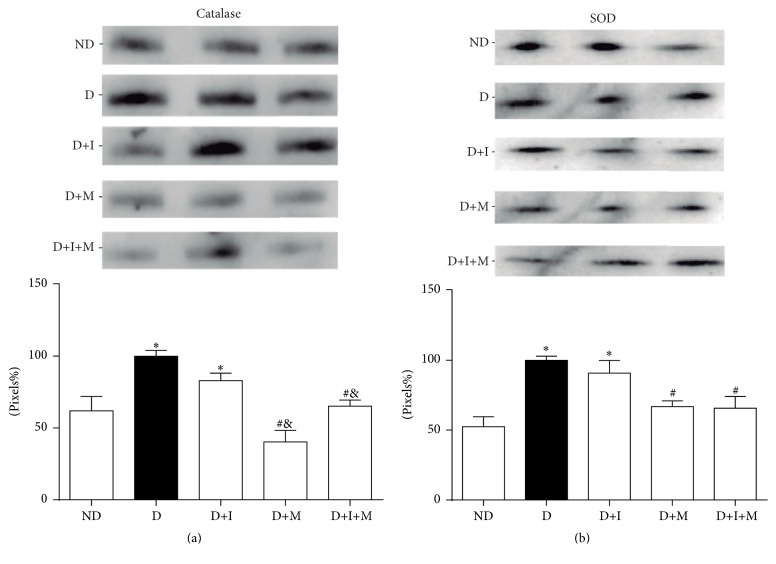
Catalase (a) and SOD (b) protein content of gastrocnemius muscle of nondiabetic and diabetic rats treated with insulin and/or metformin. Nondiabetic (ND), diabetic (D), diabetic treated with insulin (D+I), diabetic treated with metformin (D+M), and diabetic treated with insulin and metformin (D+I+M). At the top of each graph is a representative image of the western blot assay. Bands representatives of 3 rats/group (total *n* = 6 rats/group). ^*∗*^ *p* < 0.05 versus ND, ^#^ *p* < 0.05 versus D, and ^&^ *p* < 0.05 versus D+I.

Western blotting was performed to assess catalase and SOD content in gastrocnemius muscle (Figures 4(a) and 4(b)). Catalase and SOD content increased (*p* < 0.05) in D compared to ND rats. The insulin treatment (D+I) did not change catalase and SOD content compared to D rats. However, catalase and SOD content decreased (*p* < 0.05) in D+M and D+I+M compared to D rats and catalase content also decreased compared to D+I rats (Figures 4(a) and 4(b)).

## 4. Discussion

The present study demonstrated the effects of metformin treatment in the antioxidant defense system in gastrocnemius muscle of hypoinsulinemic diabetic rats. Metformin blocks the mitochondrial complex I [12, 32, 33] leading to decreased production of ROS [34] and decreasing mitochondrial oxidative capacity [35]. We have shown that metformin associated with insulin therapy decreases oxidative stress levels and CAT and SOD contents in gastrocnemius muscle compared to insulin therapy alone, in diabetic rats. In addition, the combined therapy maintained a similar glycemic control and insulin resistance, compared to D+I rats.

As expected, body weight, glycemic control, and insulin sensitivity was reduced in rats with a diabetic condition. Insulin therapy was able to reduce blood glucose levels in diabetic rats; however, the insulin sensitivity did not change. Although insulin treatment was effective at decreasing insulin resistance in a previous study [36], it is important to emphasize that the glycemic levels in our study were elevated; therefore a higher dose of insulin could promote a decrease in insulin resistance. The combined therapy of insulin plus metformin maintained glycemic control and insulin resistance similar to monotherapy with insulin. This is in agreement with unchanged HbA_1c_ after addition of metformin with continuous subcutaneous insulin therapy in type 1 diabetic patients [37]. However, the combined therapy showed an improvement in insulin sensitivity when compared to the diabetic condition, which was not observed in insulin therapy alone. This suggests that the dual therapy had unique effects that could promote greater glycemic control over time. This can be explained since metformin and insulin promote the translocation of GLUT4 by different pathways [38]. Metformin acts via 5′-AMP-activated protein kinase whereas insulin binds insulin receptor and both pathways activate AKT (AS160) which leads to a greater uptake of glucose [10].

In the present study the monotherapy with metformin was not able to reduce plasma glucose. In T2D patients with hyperinsulinemia, metformin improves glycemic control by enhancing insulin sensitivity in muscle increasing glucose uptake and in liver, decreasing the production of hepatic glucose [39]. However, in hypoinsulinemic condition the metformin alone was not able to reduce blood glucose levels. Besides this, untreated hyperglycemia can lead to insulin resistance [23]. Moreover, the rats treated with metformin alone showed a decrease in body weight compared to nondiabetic rats. The metformin activates intracellular AMPK, which reduces the activity of acetyl CoA carboxylase enzyme (ACC). The low activity of ACC decreases the biosynthesis of fatty acids and increases b-oxidation, which can promote weight loss [40].

The hyperglycemia promoted an increase in the generation of ROS [41] leading to increased activity of SOD, which was observed in diabetic rats [42, 43]. Moreover, oxidative stress, generated by hyperglycemia, activates signaling pathway JNK/FOXO [44] and the phosphorylation of the transcription factor FOXO by JNK in diabetic conditions. FOXO phosphorylated translocate to the nucleus upregulating the expression of MnSOD and the catalase gene [44, 45]. According to this, in muscle cells of diabetic animals the hyperglycemia increases SOD and catalase protein content.

Surprisingly, the metformin treatment reduced the SOD and catalase protein content. Thus, we propose that metformin should indirectly reduce the expression of these enzymes by decreasing ROS production and oxidative stress. Moreover, the blocking of mitochondrial complex I can decrease superoxide anion production in the mitochondria [46, 47], decreasing FRAP, TBARS, SOD, and catalase. We expect that these regulations can occur in monotherapy with metformin and dual therapy with insulin plus metformin. Diabetic rats treated with insulin showed similar oxidative stress levels to diabetic rats, as measured by FRAP and TBARS assays. Considering that hyperglycemia promotes an increase in oxidative stress levels, these results were unexpected. We suggest that the glycemic level is independently associated with oxidative stress levels, notably reaching a plateau when going from moderately controlled diabetes to uncontrolled diabetes. It is probably that changes in glycemia, in tight glycemic control using intensive insulin therapy, will be able to show parallel changes in oxidative stress levels.

The increase of ROS can also stimulate the peroxidase-glutathione and glutathione reductase systems in different tissues [4]. Thus, the activities of Gpx, GR, and G6PD enzymes and the concentration of GSH increased in untreated diabetic rats, suggesting an imbalance in the oxidant status. The animals treated with insulin showed the same behavior in relation to these markers, except that the GR enzymatic activity was reduced. Insulin-mediated glucose uptake could explain the reduction in GR activity, since this enzyme is negatively modulated by glucose.

Furthermore, we found similar GR activity after metformin treatment, even if the intracellular glucose was increased. This may be related to the ability of metformin to block complex I, leading to the accumulation of NADH in the mitochondrial matrix. There is evidence that, in this condition, NADH is oxidized to NAD by nicotinamide nucleotide transhydrogenase enzyme, also present in the inner mitochondrial membrane. This enzyme transfers electrons from NADH to NADP^+^, reducing it to NADPH [48, 49]. The accumulation of NADPH, the substrate for GR, can explain the absence of any change in the activity of this enzyme in all rats metformin-treated, compared to the untreated diabetic rats. It is also known that NADPH acts as a negative allosteric modulator of the G6PDH enzyme [50], decreasing its activity, as observed in monotherapy with metformin and dual therapy with insulin and metformin. Despite the treatment with metformin having altered the SOD and catalase content and showing evidence of NADP^+^ accumulation in mitochondria by decreasing G6PDH, we did not find any differences in Gpx activity or in the concentration of GSH in diabetic animals treated with metformin, compared to the untreated diabetic animals.

In the present study, the diabetic rats and insulin-treated diabetic rats showed an increase in total antioxidant capacity in muscle compared to nondiabetic rats. Some studies have shown that total antioxidant capacity is decreased in diabetic rats [51, 52], in experiments that evaluated the animals after 4 to 6 weeks. The data presented in our study, in which animals remained diabetic for two weeks, showed that there was an initial compensatory increase of the antioxidant capacity, induced as a response to an overproduction of free radicals as described in a previous study [53]. However, as shown here, metformin prevented the increase in total antioxidant capacity in both treatments, monotherapy or combined therapy with insulin. This may have occurred due to metformin action controlling the ROS production in gastrocnemius muscle of diabetic rats.

The reactive oxygen species is directly related to the increase in lipid peroxidation [54, 55]. We showed an increase in lipid peroxidation levels in gastrocnemius of untreated diabetic animals, as described in other studies [56–58]. However, the animals treated with metformin showed a decrease in the lipid peroxidation. The treatment with insulin alone did not prevent the increase of lipid peroxidation of the muscle. The lipid peroxidation in the cell membrane is associated with insulin resistance [59]; this is in agreement with our results, which did not show an increase in the insulin sensitivity of the rats treated with insulin compared to untreated rats.

Finally, our results highlight that the treatment with metformin improved the antioxidant defense system independently of glycemic control. The oxidative stress in diabetes may lead to damages in endothelial, vascular smooth muscle, and myocardial function and accelerate the development of cardiovascular disease [60]. Moreover, the endothelial function and regulation of vascular tone are impaired with consequent increases in peripheral vascular resistance and inadequate regulation of oxygen supply to the skeletal muscle, which can affect muscle function [61]. A recent study showed beneficial effects of metformin on endothelial cells, since metformin repressed oxLDL-induced oxidative stress in endothelial cells [62]. Therefore, dual therapy with metformin plus insulin could promote additional vascular benefits for the treatment of type 1 diabetic reducing the oxidative stress independently of glycemic control.

It is important to mention that this study evaluated gastrocnemius muscle which contains more type II fibers, so further studies with type I muscle fibers are warranted, given that this muscle tends to be more insulin sensitive and oxidative, and the effects of metformin treatment could be different. Furthermore, the impact of the reducing of ROS promoted by metformin treatment should also be evaluated over the long term.

## 5. Conclusion

To the best of our knowledge, this is the first report to reveal that dual therapy with metformin and insulin reduced the oxidative stress and prevented lipid peroxidation in gastrocnemius muscle of hypoinsulinemic diabetic rats when compared with the use of insulin therapy alone. Finally, these results contributes to revealing that dual therapy with metformin and insulin may be of interest for additional benefits for type 1 diabetes treatment.
